# Clinical characteristics of patients with non-tuberculous mycobacterial pulmonary disease: a seven-year follow-up study conducted in a certain tertiary hospital in Beijing

**DOI:** 10.3389/fcimb.2023.1205225

**Published:** 2023-06-22

**Authors:** Qi Liu, Jingli Du, Huiru An, Xianan Li, Donglin Guo, Jiebai Li, Wenping Gong, Jianqin Liang

**Affiliations:** ^1^ Senior Department of Tuberculosis, The 8th Medical Center of PLA General Hospital, Beijing, China; ^2^ Hebei North University, Zhangjiakou, Hebei, China

**Keywords:** non-tuberculous mycobacteria (NTM), non-tuberculous mycobacterial pulmonary disease (NTM-PD), clinical feature, drug susceptibility test (DST), lymphocyte subsets

## Abstract

**Background:**

The incidence of non-tuberculous mycobacterial pulmonary disease (NTM-PD) has increased in recent years. However, the clinical and immunologic characteristics of NTM-PD patients have received little attention.

**Methods:**

NTM strains, clinical symptoms, underlying diseases, lung CT findings, lymphocyte subsets, and drug susceptibility tests (DSTs) of NTM-PD patients were investigated. Then, the counts of immune cells of NTM-PD patients and their correlation were evaluated using principal component analysis (PCA) and correlation analysis.

**Results:**

135 NTM-PD patients and 30 healthy controls (HCs) were enrolled from 2015 to 2021 in a certain tertiary hospital in Beijing. The number of NTM-PD patients increased every year, and *Mycobacterium intracellulare* (*M. intracellulare*), *M. abscessus*, *M. avium*, and *M. kansasii* were the major pathogens of NTM-PD. The main clinical symptoms of NTM-PD patients were cough and sputum production, and the primary lung CT findings were thin-walled cavity, bronchiectasis, and nodules. In addition, we identified 23 clinical isolates from 87 NTM-PD patients with strain records. The DST showed that almost all of *M. abscessus* and *M. avium and* more than half of the *M. intracellulare* and *M. avium* complex groups were resistant to anti-tuberculosis drugs tested in this study. *M. xenopi* was resistant to all aminoglycosides. *M. kansasii* was 100% resistant to kanamycin, capreomycin, amikacin, and para-aminosalicylic acid, and sensitive to streptomycin, ethambutol, levofloxacin, azithromycin, and rifamycin. Compared to other drugs, low resistance to rifabutin and azithromycin was observed among NTM-PD isolates. Furthermore, the absolute counts of innate and adaptive immune cells in NTM-PD patients were significantly lower than those in HCs. PCA and correlation analysis revealed that total T, CD4^+^, and CD8^+^ T lymphocytes played an essential role in the protective immunity of NTM-PD patients, and there was a robust positive correlation between them.

**Conclusion:**

The incidence of NTM-PD increased annually in Beijing. Individuals with bronchiectasis and COPD have been shown to be highly susceptible to NTM-PD. NTM-PD patients is characterized by compromised immune function, non-specific clinical symptoms, high drug resistance, thin-walled cavity damage on imaging, as well as significantly reduced numbers of both innate and adaptive immune cells.

## 1 Introduction

Non-tuberculosis mycobacteria (NTM) is a member of the Mycobacterium genus other than the Mycobacterium tuberculosis complex (such as M. tuberculosis, M. bovis, M. africanum, M. microti, M. canetti, M. caprae, and M. pinnipedii) and M. leprae (Sharma and Upadhyay, 2020). NTM has different characteristics from M. tuberculosis, such as being more sensitive to acids and alkalis, generally resistant to anti-tuberculosis (TB) drugs, and grows at less stringent temperatures than M. tuberculosis (Falkinham, 2002; Mortaz et al., 2014; Zhou et al., 2019). In addition, most NTM strains are naturally resistant to first- and second-line anti-TB drugs, resulting in poor therapeutic efficacy after NTM infection, which causes prolonged suffering and a high economic burden (Diel et al., 2017).

NTMs are widely distributed in water, soil, and dust, and can cause infections of the lungs, skin, bones, joints, lymph nodes, and other extrapulmonary tissues, of which non-tuberculous mycobacterial pulmonary disease (NTM-PD) is the most common (Jeon, 2019). It has been reported that NTMs have become an important cause of zoonotic tuberculosis (Clarke et al., 2022; Tingan et al., 2022), and some NTM species have potential for causing disease in animals and humans, especially in low-income countries (Katale et al., 2014). Therefore, the “One Health” strategy should be considered for TB prevention and control (Erkyihun and Alemayehu, 2022).

The prevalence of NTM-PD is increasing worldwide, with recent reports estimating a prevalence of 2.3-6.5 per 100,000 in Europe (Prevots and Marras, 2015; Ringshausen et al., 2016; van der Laan et al., 2022). In Japan, prevalence rates were even higher at an estimated 33-65 cases per 100,000, and in the United States, incidence rates were 3.1 per 100,000 in 2008, increasing to 4.7 per 100,000 in 2015 (Morimoto et al., 2014; Winthrop et al., 2020; Schildkraut et al., 2021). In China, overall epidemiologic information on NTM-PD is still not available. However, according to a prospective surveillance study conducted in China, the proportion of NTM-PD in PTB patients showed significant geographical variation, ranging from 3.2% in the northwest to 9.2% in the south (Tan et al., 2021). The most common species was *M. intracellulare*, followed by *M. abscessus* complex (Tan et al., 2021).

Currently, NTM-PD has become a public health problem. The treatment of NTM-PD is usually based on the results of mycobacterial species identification, drug susceptibility test (DST), and the guidelines’ recommendations for diagnosing and treatment of NTM-PD (Association., T.B.o.C.M, 2020; Daley et al., 2020). Unlike *M. tuberculosis*, there are differences in the types of drugs and regimens used to treat NTM-PD caused by different NTM strains, as well as differences in treatment success rates. For example, treatment success rates in patients with *M. kansasii* (89.9%) were significantly higher than those for *M. avium* complex (MAC, 65.0%) and *M. abscessus* (36.1%) (Cheng et al., 2022).

Furthermore, previous studies have reported several risk factors for the development of NTM-PD, such as age, sex, body mass, interstitial lung disease, bronchiectasis, bronchiolitis, postoperative pulmonary complications, and neoadjuvant/adjuvant treatments (Park et al., 2020). In China, studies on NTM-PD mainly focused on populations in southern and coastal China (Tan et al., 2018; Hu et al., 2019; Xu et al., 2019; Lou et al., 2023), but studies on the epidemiologic and clinical characteristics of NTM-PD in people in northern regions, especially in Beijing, are still lacking. Therefore, this retrospective study was conducted to obtain first-hand information on the incidence, clinical and immunologic characteristics of patients with NTM-PD and drug resistance rates of NTM strains in Beijing from January 2015 to December 2021. This study will provide data for hospital and government authorities to understand the epidemiologic characteristics of NTM-PD and highlight a reference for determining NTM-PD prevention and treatment strategies in a “One Health” perspective.

## 2 Materials and methods

### 2.1 Study design and ethics statement

This retrospective study was performed on inpatients at the Eighth Medical Center of PLA General Hospital and approved by the Ethics Committee of the Eighth Medical Center of PLA General Hospital. Furthermore, the protocol of this study was conducted according to the ethical standards of the Declaration of Helsinki.

### 2.2 Study subjects

NTM-PD patients with a positive acid-fast staining on sputum, positive bronchoalveolar lavage fluid smear, or positive culture of mycobacterial P-nitro benzoic acid (PNB) were enrolled. The NTM-PD was diagnosed by consulting the medical records system and combining clinical symptoms and lung CT findings. NTM diagnosis was made according to a previous study (Daley et al., 2020). Moreover, a healthy control (HC) group consisting of 30 healthy individuals was incorporated in the analysis of T lymphocyte subsets. There was no significant difference in age and gender between the HCs and NTM-PD groups. The individuals in HC group with the following diseases will be excluded, including HIV, malignant tumors, immune disorders, blood system diseases, TB, chronic obstructive pulmonary disease (COPD), diabetes mellitus (DM), and other factors affecting cellular immunity as well as the underlying diseases.

### 2.3 Mycobacteria culture and identification of NTM

#### 2.3.1 Traditional mycobacteria culture and identification of NTM

As outlined in the “Testing Procedures for Tuberculosis Laboratory” (Zhao and Pang, 2015), sputum samples that tested positive for acid-fast staining were inoculated into Lowenstein-Jensen (L-J) culture medium, and identified using PNB and thiophene-2-hydroxy-1-hydrazine. Mycobacterium strains that grew within seven days were considered fast-growing, while those that took more than seven days to grow were classified as slow-growing mycobacteria. If a strain can grow in the culture medium after PNB addition, it will be identified as NTM.

#### 2.3.2 Molecular identification of NTM species

*M. tuberculosis* (MTB) and NTM were identified using the Diagnostic Kit for MTB/NTM DNA kit with a PCR-fluorescence probe method (CapitalBio Technology, Beijing, China), as described in our previous study (Liang et al., 2021). Briefly, sputum samples were collected from each patient and mixed with an equal amount of 4% NaOH solution in a centrifuge tube. After incubation at room temperature for 30 minutes, the sputum was centrifuged at 12,000 rpm for 5 minutes. The supernatant was discarded, and 1 mL of normal saline was added. The tube was centrifuged again under the same conditions, and the supernatant was discarded. 50 μL of nucleic acid extraction solution was added, and the mixture was shaken using a DNA extractor for 5 minutes. The extracted nucleic acid was heated at 95 °C for 5 minutes, and then added to the PCR reaction system. The amplification program was run at 37 °C for 300s, 94 °C for 180 s, 94 °C for 15 s, and 60 °C for 30 s, for a total of 40 cycles. The FAM and VIC channels were used for detection, and fluorescent spots were collected at 60 °C for 30 s. A Ct value of less than 40 was considered positive, and a non-sigmoid amplification curve or a Ct value of 40 was considered negative. The operation procedures and results interpretation were carried out according to the manufacturer’s instructions.

To further identify positive PNB samples initially determined through traditional mycobacterial culture, NTM were subjected to identification using the Mycobacterial Species Identification Kit (CapitalBio Technology, Beijing, China) in accordance with the manufacturer’s instructions.

### 2.4 Drug susceptibility test

The drug susceptibility of NTM strains was determined using a Minimal Inhibitory Concentration (MIC) Drug Susceptibility Test Kit for Mycobacteria (Encode Medical Engineering Co., Ltd, Zhuhai, Guangdong, China) following the manufacturer’s instructions, “Testing Procedures for Tuberculosis Laboratory” (Zhao and Pang, 2015), and “Susceptibility Testing of Mycobacteria, Nocardia spp., and Other Aerobic Actinomycetes” (CLSI, 2011). This Drug Susceptibility Test Kit contained 12 drugs, including isoniazid (INH), rifampicin (RFP), ethambutol (EMB), streptomycin (SM), levofloxacin (LVFX), amikacin (AMK), kanamycin (KM), capreomycin (CPM), para-aminosalicylic acid (PAS), rifabutin (RFB), rifapentine (RFT), and azithromycin (AZM). In brief, the sterile diluent of 3 mL was added to the lyophilized antimicrobial agent inhibitor, followed by thorough mixing. A volume of 100 μl of this solution was added to 11 mL of the susceptibility test medium and mixed well. A volume of 180 μl of the susceptibility test medium was added to the wells of a microtiter plate as the control. Several colonies were picked from a solid Lowenstein-Jensen (L-J) culture medium and ground to a suspension with an optical density of 1 mg/mL. A volume of 100 μl of the prepared suspension was added to the susceptibility test medium and mixed well. A volume of 200 µl of the resulting inoculated medium was added to each well of a 96-well microtiter plate, and the plate was then sealed and incubated at 37°C for 7 to 14 days. The growth of bacterial colonies was observed visually, or using a YK-909 mycobacterial susceptibility test analyzer (Encode Medical Engineering Co., Ltd, Zhuhai, Guangdong, China). The MIC of each NTM strain was determined based on the lowest concentration that showed inhibition of bacterial growth, with reference to the growth of control colonies. If the control colonies did not grow well, further incubation was performed for up to 21 days.

### 2.5 Analysis of absolute lymphocyte subsets

To exclude the influence of other diseases on the number of immune cells in patients with NTM-PD as much as possible, we only included patients with NTM-PD alone or NTM-PD with bronchiectasis, and NTM-PD patients with any other diseases were excluded. As a result, 44 NTM-PD patients and 30 healthy volunteers were selected for absolute counts analysis of lymphocyte subsets. First, the blood samples collected from NTM-PD patients and HCs were placed in heparin anticoagulant tubes. Then, 50 μL of the blood sample was transferred to a new BD TruCount tube (BD, San Jose, California, USA) containing 20 μL of the 6-color TB NK Reagent. Flow cytometry was used to detect CD45 cells labeled with percp-cy5.5 fluorescence (BD Biosciences, San Jose, CA, USA), CD3 cells labeled with FITC fluorescence (BD Biosciences, San Jose, CA, USA), CD4 cells labeled with pe-cy7 fluorescence (BD Biosciences, San Jose, CA, USA), CD8 cells labeled with APC-cy7 fluorescence (BD Biosciences, San Jose, CA, USA), CD16 cells and CD56 cells labeled with PE fluorescence (BD Biosciences, San Jose, CA, USA), and CD19 cells labeled with APC fluorescence (BD Biosciences, San Jose, CA, USA). After incubation at room temperature for 15 min in the dark, 1 × red blood cell lysate (BD, San Jose, CA, USA) was added to the samples and incubated in the dark for 15 min. Subsequently, FACS Aria II from Becton Dickinson (BD, California, USA) was used to determine the absolute counts of total T lymphocytes, CD4^+^ T lymphocytes, CD8^+^ T lymphocytes, NK cells, NK-like T (NKT) lymphocytes, and total B lymphocytes. Finally, the data were analyzed using FACS DIVA software.

### 2.6 Statistical methods

Data were analyzed using the GraphPad Prism software 9.5.1 version (San Diego, CA, USA). Statistical analysis of count data was conducted using either the chi-square test or Fisher’s exact test, depending on the sample size. The absolute counts of lymphocyte subsets between patients with NTM-PD and healthy individuals were compared with an Unpaired *t*-test or nonparametric test (Mann-Whitney test) according to the normality, and *P* < 0.05 was considered statistically different. The normality of data was tested by the Shapiro-Wilk test and Kolmogorov-Smirnov test, and *P* > 0.05 was considered that the data conformed to a normal distribution. The data were shown as mean ± standard error of the mean (SEM). Furthermore, a principal component analysis (PCA) was performed to analyze the principal component of absolute counts of lymphocyte subsets. Finally, the correlation of these components was determined by the Pearson’s *r* method.

## 3 Results

### 3.1 Baseline characteristics

**Figure 1:**
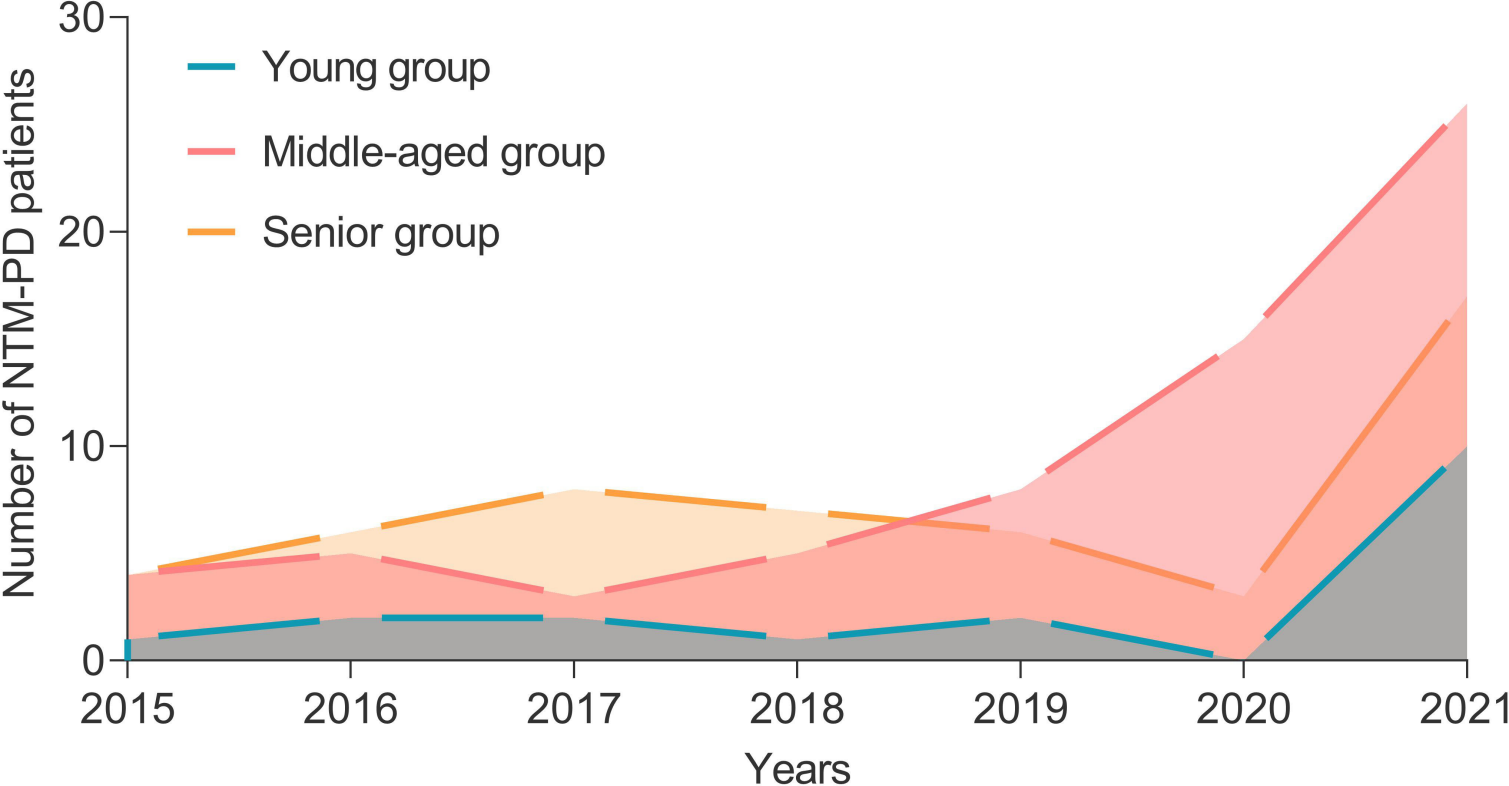
The number of patients with NTM-PD in a hospital from 2015 to 2021.

A total of 135 inpatients with NTM-PD and 30 healthy individuals from the physical examination center were enrolled in this study. From 2015 to 2021, the number of patients with NTM-PD was 9, 13, 13, 13, 16, 18, and 53, respectively, with an increasing trend each year (**Figure 1**). These patients with NTM-PD were divided into three groups according to age: young (20-45 years, 17 cases), middle-aged (45-65 years, 67 cases), and elderly (≥65 years, 51 cases). Furthermore, middle-aged and elderly patients accounted for 87.41% (118 cases) of the total cases, of which 58.52% (79 cases) were male (**Table 1**). Remarkably, we observed noteworthy gender disparities among NTM-PD patients in the senior and middle-aged groups, evidenced by a markedly lower proportion of females in the former group (**Table 1**, *P* = 0.0242).

**Table 1:** The composition ratio of NTM-PD patients in various age groups by sex (n, %).

Age Group^*^/Gender	Male	Female	Total	*P* value (Fisher’s exact test)
Young group	9 (6.67%)	8 (5.92%)	17 (12.59%)	Reference
Middle-aged group	34 (25.19%)	33 (24.44%)	67 (49.63%)	>0.9999
Senior group	36 (26.67%)	15 (11.11%)	51 (37.78%)	0.2390 (Senior vs. Young) **0.0242 (Senior vs. Middle)**
Total	79 (58.52%)	56 (41.48%)	135 (100%)	

^*^ The patients with NTM-PD were divided into three groups, including the young (20-45 years old), the middle-aged (45-65 years old), and the elderly (≥65 years old) groups.Bold values means P < 0.05.

### 3.2 Identification of NTM species based on the medical visit records

**Figure 2:**
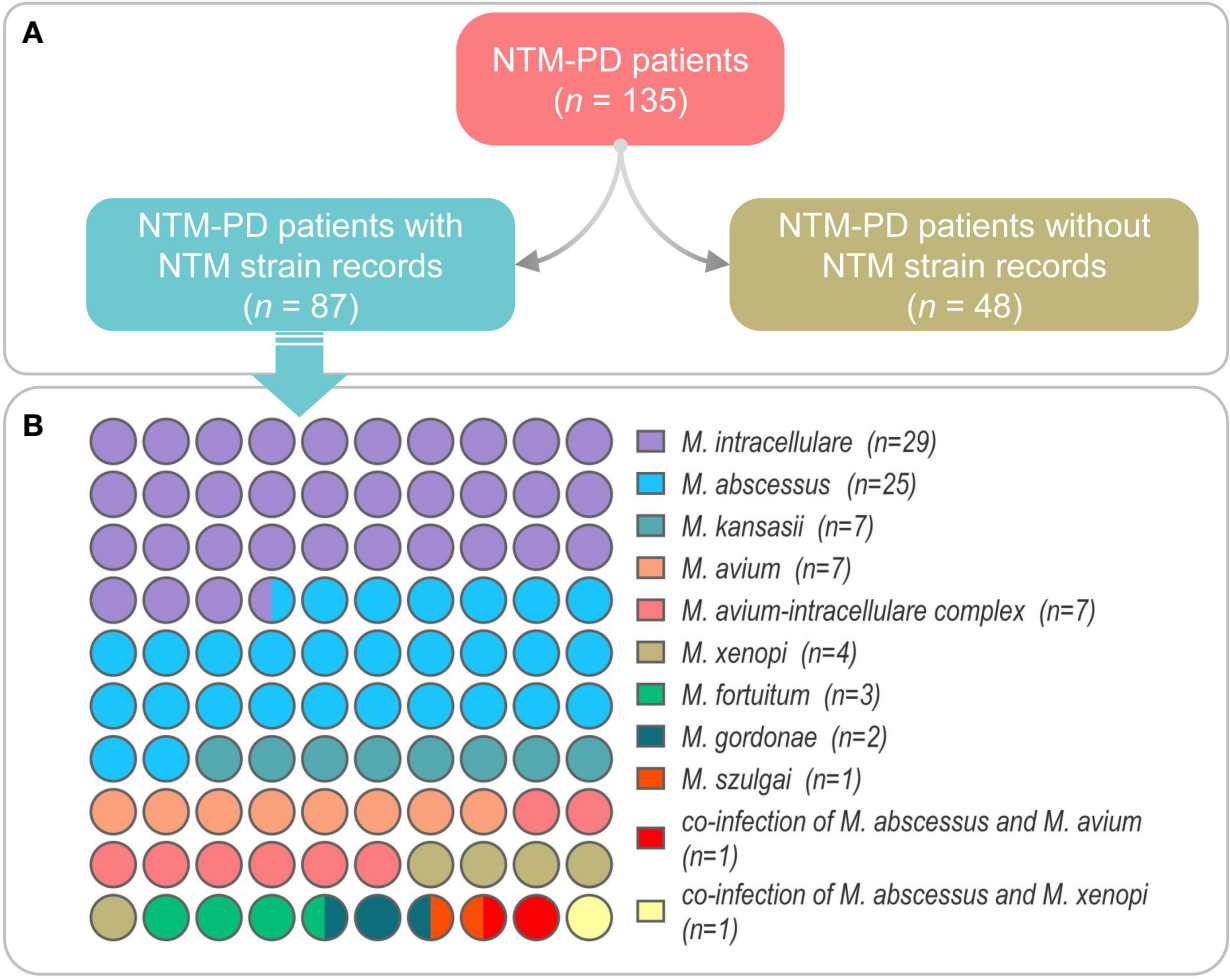
The NTM strain information was obtained from the medical records of NTM-PD patients **(A)** and the number of NTM-PD patients in each NTM species **(B)**. Co-infection is defined as the presence of two or more NTM strains infecting a single NTM-PD patient simultaneously.

In this study, we retrospectively reviewed the medical visit records of 135 patients with NTM-PD. Among them, there were 26 cases of fast-growing non-tuberculous mycobacterium lung disease and 109 cases of slow-growing non-tuberculous mycobacterium lung disease. We found that only 87 patients had specific NTM strain records of species, and the other 48 patients had no NTM strain records queried. Therefore, we further investigated the NTM species of these 87 patients with NTM-PD (**Figure 2A**). As a result, we identified *M. intracellulare* in 29 patients, *M. abscessus* in 25 patients, *M. kansasii* in 7 patients, *M. avium* in 7 patients, *M. avium*-*intracellulare* complex in 7 patients, *M. xenopi* in 4 patients, *M. fortuitum* in 3 patients, *M. gordonae* in 2 patients, *M. szulgai* in 1 patient, co-infection of *M. abscessus and M. avium* in 1 patient, and co-infection of *M*. *abscessus and M*. *xenopi* in 1 patient (**Figure 2B**).

### 3.3 Basic disease of 87 NTM-PD patients with NTM species records

We further investigated the primary diseases of the 87 NTM-PD patients with NTM species records. The results showed that 21 cases had bronchiectasis (24.1%), 15 cases had chronic obstructive pulmonary disease (COPD) (17.2%), 11 cases had cardiovascular disease (CVD, 12.6%), nine cases had neoplastic disease (ND, 10.3%), eight cases had interstitial pneumonia (IP, 9.2%), eight cases had TB (9.2%), seven cases had diabetes mellitus (DM, 8.0%), four cases had reflux esophagitis (RE, 4.6%), four cases had immune system disease (ISD, 4.6%), and nine cases had no underlying disease (NUD, 10.3%) (**Table 2**).

**Table 2:** Primary diseases of 87 NTM-PD patients with NTM strains records (*n*, %).

NTM strains (*n*)	Bronchiectasis	COPD	CVD	ND	IP	PTB	DM	RE	ISD	NUD
*M. intracellulare* (29)	5 (5.7)	8 (9.2)	1 (1.1)	3 (3.4)	4 (4.6)	2 (2.3)	1 (1.1)	2 (2.3)	0 (0)	3 (3.4)
*M. abscessus* (25)	5 (5.7)	1 (1.1)	5 (5.7)	3 (3.4)	3 (3.4)	0 (0)	2 (2.3)	1 (1.1)	2 (2.3)	3 (3.4)
*M. kansasii* (7)	2 (2.3)	0 (0)	3 (3.4)	0 (0)	1 (1.1)	1 (1.1)	1 (1.1)	0 (0)	0 (0)	0 (0)
*M. avium* (7)	3 (3.4)	0 (0)	0 (0)	3 (3.4)	0 (0)	0 (0)	0 (0)	0 (0)	0 (0)	1 (1.1)
*M. avium* - *intracellulare* complex (7)	4 (4.6)	1 (1.1)	0 (0)	0 (0)	0 (0)	1 (1.1)	0 (0)	0 (0)	0 (0)	1 (1.1)
*M. xenopi* (4)	0 (0)	4 (4.6)	0 (0)	0 (0)	0 (0)	0 (0)	1 (1.1)	1 (1.1)	1 (1.1)	0 (0)
*M. gordonae* (3)	0 (0)	0 (0)	0 (0)	0 (0)	0 (0)	2 (2.3)	0 (0)	0 (0)	0 (0)	1 (1.1)
*M. fortuitum* (2)	2 (2.3)	0 (0)	1 (1.1)	0 (0)	0 (0)	1 (1.1)	1 (1.1)	0 (0)	0 (0)	0 (0)
*Co-infection* ^*^ *of M. abscessus* and *M. avium* (1)	0 (0)	0 (0)	1 (1.1)	0 (0)	0 (0)	1 (1.1)	0 (0)	0 (0)	0 (0)	0 (0)
*Co-infection* ^*^ *of M. abscessus and xenopi* (1)	0 (0)	1 (1.1)	0 (0)	0 (0)	0 (0)	0 (0)	1 (1.1)	0 (0)	0 (0)	0 (0)
*M. szulgai* (1)	0 (0)	0 (0)	0 (0)	0 (0)	0 (0)	0 (0)	0 (0)	0 (0)	1 (1.1)	0 (0)
Total (RMB)	21 (24.1)	15 (17.2)	11 (12.6)	9 (10.3)	8 (9.2)	8 (9.2)	7 (8.0)	4 (4.6)	4 (4.6)	9 (10.3)

^*^, Co-infection is defined as the presence of two or more NTM strains infecting a single NTM-PD patient simultaneously.

COPD, chronic obstructive pulmonary; CVD, cardiovascular disease; IP, interstitial pneumonia; ISD, immune system disease; ND, neoplastic diseases; PTB, Pulmonary tuberculous; RE, reflux esophagitis; NUD, no underlying disease.

### 3.4 Clinical symptoms of 87 NTM-PD patients with NTM species records

The clinical symptoms of 87 NTM-PD patients with NTM species records were observed and recorded. The results showed that: 1) 78 cases had cough and sputum (89.7%), mainly caused by *M. avium* (100%), *M. intracellulare* (93.1%), and *M. abscessus* (92%); 2) Hemoptysis was observed in 26 cases (29.9%), 50% of whom were infected with *M. xenopi*; 3) Fever, chest tightness, and shortness of breath were observed in 14 cases (16.1%), 66.7% and 24.1% of which were infected with *M. fortuitum* and *M. intracellulare*, respectively (**Table 3**).

**Table 3:** Clinical symptoms of 87 NTM-PD patients with NTM species records.

NTM strains	Cough and phlegm (*n*, %)	Hemoptysis (*n*, %)	Heating (*n*, %)	Chest tightness and short breath (*n*, %)
*M. intracellulare* (29)	27 (93.1%)	9 (31.0%)	7 (24.1%)	7 (24.1%)
*M. abscessus* (25)	23 (92.0%)	8 (32.0%)	2 (8.0%)	3 (12%)
*M. avium* (7)	7 (100%)	2 (28.6%)	1 (14.3%)	0 (0)
*M. avium-intracellulare* complex (7)	6 (85.7%)	1 (14.3%)	0 (0)	0 (0)
*M. kansasii* (7)	5 (71.4%)	1 (14.3%)	2 (28.6%)	1 (14.3%)
*M. xenopi* (4)	3 (75%)	2 (50.0%)	1 (25.0%)	0 (0)
*M. fortuitum* (3)	2 (66.7%)	1 (33.3%)	0 (0)	2 (66.7%)
*M. gordonae* (2)	2 (100%)	0 (0)	0 (0)	0 (0)
*M. szulgai* (1)	1 (100%)	0 (0)	0 (0)	0 (0)
*Co-infection* ^*^ *of M. abscessus and M. avium* (1)	1 (100%)	1 (100%)	0 (0)	1 (100%)
*Co-infection* ^*^ *of M. abscessus and M. xenopi* (1)	1 (100%)	1 (100%)	1 (100%)	0 (0)
Total 87	78 (89.7%)	26 (29.9%)	14 (16.1%)	14 (16.1%)

^*^, Co-infection is defined as the presence of two or more NTM strains infecting a single NTM-PD patient simultaneously.

### 3.5 Lung imaging findings of 87 NTM-PD patients with NTM species records

The imaging findings were classified according to “Expert Consensus 2021 on the Imaging Diagnosis of Non-tuberculous Mycobacteria Pulmonary Disease” (Budong et al., 2021). Among 87 NTM-PD patients with NTM species records, 33 cases were accompanied by cavitary pulmonary lesions (33/87, 37.9%). Among these 33 cases, three cases (9.10%) had thick-walled cavitary lesions with *M. intracellulare* pulmonary disease (PD), and the other 30 cases (90.91%) had thin-walled cavitary lesions. Furthermore, 42 cases had predominantly bronchiectasis and nodules in the lung, accounting for 48.3% (42/87), and 12 were consolidating lesion types, accounting for 13.79% (12/87). In addition, one case with PD infected by *M. xenopi* and two cases infected by *M. gordonae* were characterized by pulmonary lesion type of bronchiectasis and nodules predominantly, accompanied by swollen lymph nodes of mediastinal simultaneously. We also found two cases infected with *M. kansasii*, one with *M. intracellulare*, and one with *M. fortuitum*, accompanied by a small amount of exudative pleural effusion (**Table 4**).

**Table 4:** Lung CT findings of 87 NTM-PD patients with NTM species records.

NTM (*n*)	Cavity-dominated type (*n*, %) ^a^	Bronchiectasis and nodules-dominated type (*n*, %) ^b^	Consolidation-dominated type (*n*, %) ^c^
*M. intracellulare* (29)	17 (58.6%)	10 (34.5%)	2 (6.9%)
*M. abscessus* (25)	7 (28%)	13 (52%)	5 (20%)
*M. avium* (7)	1 (14.3%)	5 (71.4%)	1 (14.3%)
*M. avium-intracellulare* complex (7)	2 (28.6%)	5 (71.4%)	0 (0)
*M. kansasii* (7)	2 (28.6%)	2 (28.6%)	3 (42.9%)
*M. xenopi* (4)	1 (25%)	2 (50%)	1 (25%)
*M. fortuitum* (3)	0 (0)	3 (100%)	0 (0)
*M. gordonae* (2)	0 (0)	2 (100%)	0 (0)
*M. szulgai* (1)	1 (100%)	0 (0)	0 (0)
*Co-infection ^d^ of M. abscessus and M. avium* (1)	1 (100%)	0 (0)	0 (0)
*Co-infection ^d^ of M. abscessus and M. xenopi* (1)	1 (100%)	0 (0)	0 (0)
Total 87	33 (37.9)	42 (48.3)	12 (13.8)

a , Cavity-dominated type means cavity is predominant in the lung;

b , Bronchiectasis and nodules-dominated type, the main focus in the lung is bronchiectasis or the coexistence of bronchiectasis and nodules.

c , consolidation-dominated type, consolidation occurs predominantly in the lung.

d , Co-infection is defined as the presence of two or more NTM strains infecting a single NTM-PD patient simultaneously.

### 3.6 DST of NTM strains

Out of the 87 NTM-PD patients with NTM species records, only DST results of 23 NTM strains were available. The DST results of these 23 NTM strains were further investigated in this study and presented in **Table 5**. The results showed that most of the NTM strains had resistance to KM and CPM (87%, 20/23). Additionally, the majority of strains were also resistant to AMK and PAS (82.6%) and INH (73.9%). Almost two-thirds of the strains were resistant to RFP, SM, EMB, LVFX, and RFT (ranging from 65.2% to 69.6%). Furthermore, 47.8% of the strains were resistant to AZM, and 34.8% of the strains were resistant to RFB.

**Table 5:** The resistance of 23 NTM strains to 12 TB drugs detected by DST (*n*, %).

Drugs	*M. intracellulare* (*n* = 7)	*M. abscessus* (*n* = 6)	*M. kansasii* (*n* = 3)	*M. avium* (*n* = 3)	*M. xenopi* (*n* = 2)	*M. avium-intracellulare* complex (*n* = 2)	Total (*n* = 23)
INH	5 (71.4%)	6 (100%)	1 (33.3%)	3 (100%)	1 (50%)	1 (50%)	17 (73.9%)
SM	4 (57.1%)	6 (100%)	0 (0)	3 (100%)	2 (100%)	1 (50%)	16 (69.6%)
EMB	5 (71.4%)	6 (100%)	0 (0)	3 (100%)	1 (50%)	1 (50%)	16 (69.6%)
RFP	4 (57.1%)	6 (100%)	0 (0)	3 (100%)	2 (100%)	1 (50%)	16 (69.6%)
LVFX	4 (57.1%)	6 (100%)	0 (0)	3 (100%)	1 (50%)	1 (50%)	15 (65.2%)
KM	5 (71.4%)	6 (100%)	3 (100%)	3 (100%)	2 (100%)	1 (50%)	20 (87%)
CPM	5 (71.4%)	6 (100%)	3 (100%)	3 (100%)	2 (100%)	1 (50%)	20 (87%)
AMK	4 (57.1%)	6 (100%)	3 (100%)	3 (100%)	2 (100%)	1 (50%)	19 (82.6%)
PAS	4 (57.1%)	6 (100%)	3 (100%)	3 (100%)	2 (100%)	1 (50%)	19 (82.6%)
RFT	3 (42.9%)	6 (100%)	0 (0)	3 (100%)	2 (100%)	1 (50%)	15 (65.2%)
RFB	1 (14.3%)	4 (66.7%)	0 (0)	1 (33.3%)	1 (50%)	1 (50%)	8 (34.8%)
AZM	2 (28.6%)	5 (83.3%)	0 (0)	2 (66.7%)	1 (50%)	1 (50%)	11 (47.8%)

AMK, Amikacin; AZM, azithromycin; CPM, Capreomycin; EMB, Ethambutol; INH, Isoniazid; KM, Kanamycin; LVFX, Levofloxacin; PAS, Para-aminosalicylic acid; RFB, Rifabutin; RFP, Rifampicin; RFT, Rifapentine; SM, Streptomycin.

Among the 23 strains identified by DST, we found that (**Table 5**): 1) *M. intracellulare*: Among the seven strains identified, five were resistant to INH, EMB, KM, and CPM, four were resistant to SM, RFP, LVFX, AMK, and PAS, three were resistant to RFT, two were resistant to AZM, and one was resistant to RFB; 2) *M. abscessus*: All six strains identified were resistant to INH, SM, EMB, RFP, LVFX, KM, CPM, AMK, PAS, and RFT, four were resistant to RFB, and five were resistant to AZM; 3) *M. kansasii*: All three strains identified were resistant to KM, CPM, AMK, and PAS, and one was resistant to INH; 4) *M. avium*: All three strains identified were resistant to INH, SM, EMB, RFP, LVFX, KM, CPM, AMK, PAS, and RFT, two were resistant to AZM, and one was resistant to RFB; 5) *M. xenopi*: Both strains identified were resistant to SM, RFP, KM, CPM, AMK, PAS, and RFT, and one was resistant to INH, EMB, LVFX, RFB, and AZM; 6) *M. avium-intracellulare* complex: One strain was resistant to all tested drugs (Association., T.B.o.C.M, 2020; Daley et al., 2020)

### 3.7 Comparison of absolute counts of T lymphocytes in patients with NTM-PD and HCs

**Figure 3:**
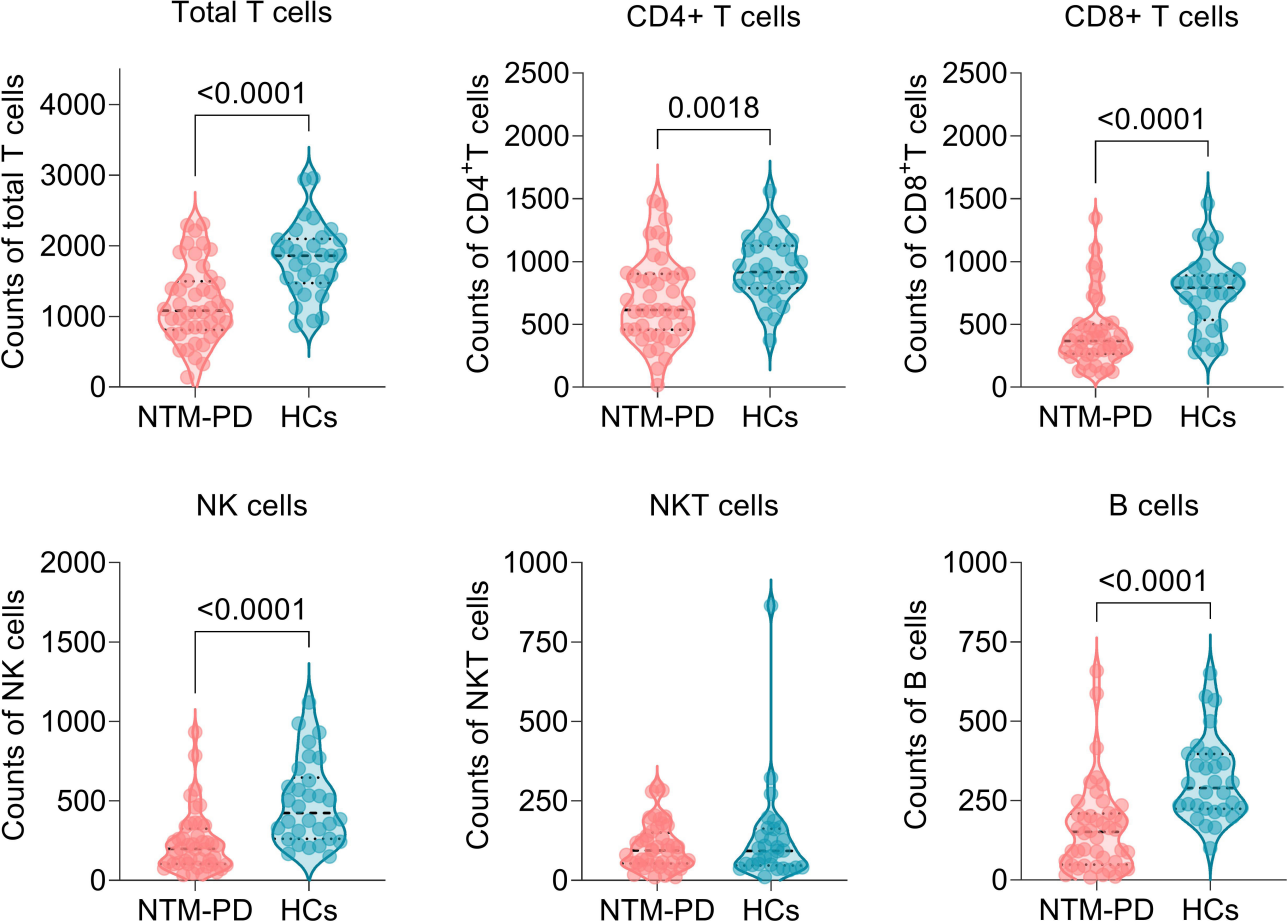
Comparison of absolute counts of total T lymphocytes, CD4^+^ T lymphocytes, CD8^+^ T lymphocytes, NK cells, NKT cells, and B cells between NTM-PD patients and HCs.

To evaluate the immune characteristics between patients with NTM-PD and healthy volunteers, we determined the absolute counts of T lymphocytes in 44 patients with NTM-PD and 30 HCs. Significantly, the patients with NTM-PD included in this experiment had no primary diseases such as HIV, malignant tumors, immune diseases, blood system diseases, TB, severe COPD, DM, and other factors affecting cellular immunity. Our results showed that the absolute counts of total T lymphocytes (*P* < 0.0001), CD4^+^ T lymphocytes (*P* = 0.0018), CD8^+^ T lymphocytes (*P* < 0.0001), NK cells (*P* < 0.0001), and B cells (*P* < 0.0001) in patients with NTM-PD were significantly lower than those in HCs (**Figure 3**). However, we observed no difference in absolute NKT cell counts between patients with NTM-PD and HCs (**Figure 3**).

**Figure 4:**
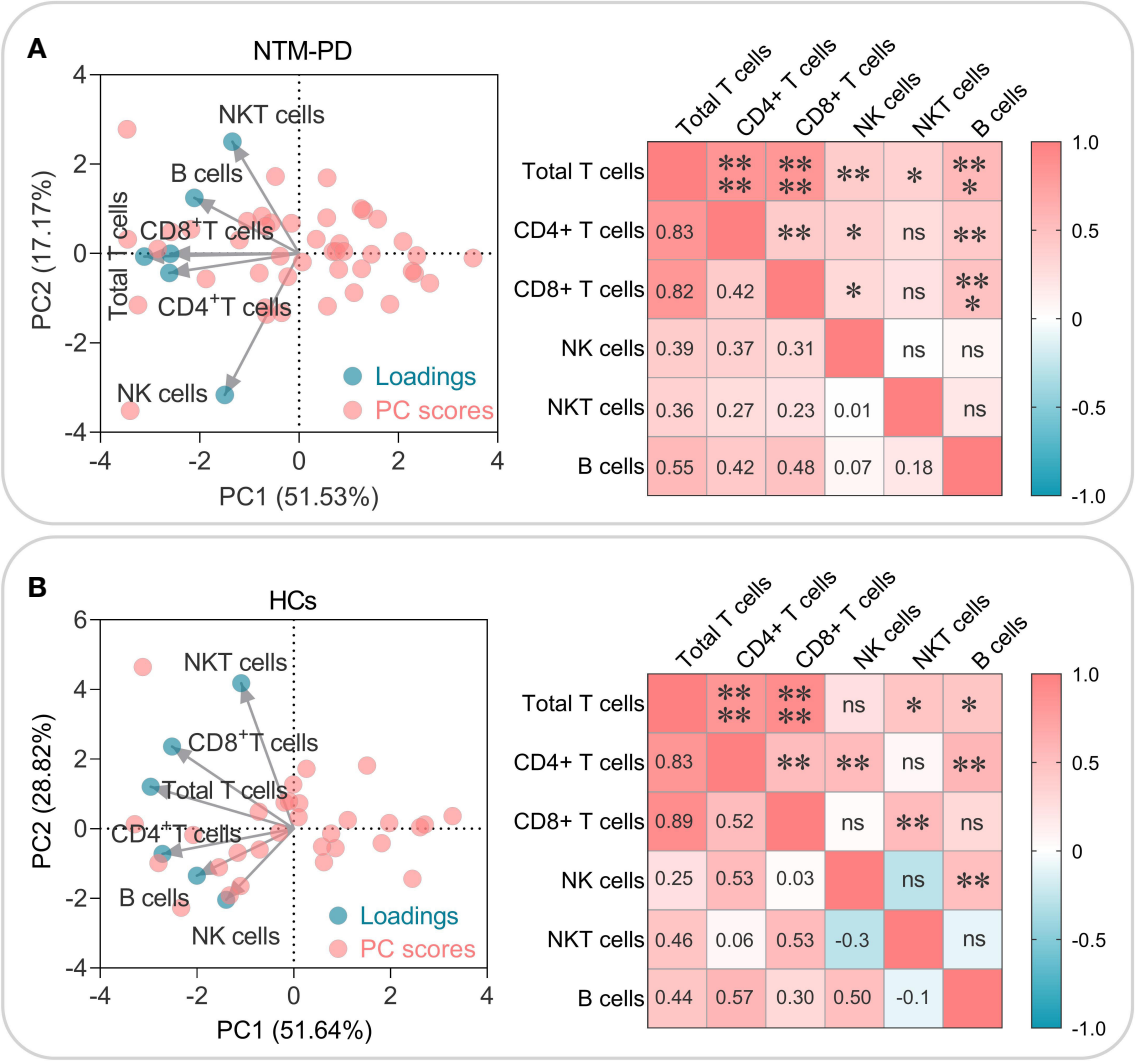
PCA and correlation analysis of the absolute counts of total T lymphocytes, CD4^+^ T lymphocytes, CD8^+^ T lymphocytes, NK cells, NKT cells, and B cells in NTM-PD patients **(A)** and HCs **(B)**. The Biplot represents the cell types correlating to the respective blue circle loadings, while the red circle shows the PC scores. The correlation plot highlights the R-value in the lower left half, and the *P*-value in the upper right half. Significance was considered at *P* < 0.05, with the levels of significance indicated as follows: *, *P* < 0.05; **, *P* < 0.01; ***, *P* < 0.001; ****, *P* < 0.0001, and ns indicating no statistical significance.

Furthermore, we also investigated the correlation among absolute counts of total T lymphocytes, CD4^+^ T lymphocytes, CD8^+^ T lymphocytes, NK cells, NKT cells, and B cells in patients with NTM-PD or HCs. Principal component analysis (PCA) and correlation analysis showed that: (1) In patients with NTM-PD (**Figure 4A**), CD4^+^ T lymphocytes, CD8^+^ T lymphocytes, and total T lymphocytes had a significant and similar role in PC1 (51.53%) and PC2 (17.17%), as they were closely clustered together. On the other hand, NKT lymphocytes and NK cells were relatively dispersed. Correlation analysis confirmed that the absolute counts of CD4^+^ T lymphocytes, CD8^+^ T lymphocytes, and total T lymphocytes were significantly correlated. In patients with NTM-PD (**Figure 4A**), there were significant positive correlations between the absolute counts of total T lymphocytes and the absolute counts of CD4^+^ T lymphocytes, CD8^+^ T lymphocytes, NK cells, NKT lymphocytes, and B lymphocytes. Furthermore, the absolute counts of CD4^+^ T lymphocytes were significantly correlated with the absolute counts of CD8^+^ T lymphocytes, NK cells, and B lymphocytes, while the absolute counts of CD8^+^ T lymphocytes had significant positive correlations with both NK cells and B lymphocytes (**Figure 4A**). (2) In HCs (**Figure 4B**), CD4^+^ T lymphocytes, CD8^+^ T lymphocytes, and total T lymphocytes displayed a similar clustering tendency. For HCs (**Figure 4B**), absolute counts of total T lymphocytes were significantly correlated with the absolute counts of CD4^+^ T lymphocytes, CD8^+^ T lymphocytes, NKT lymphocytes, and B lymphocytes. Also, the absolute counts of CD4^+^ T lymphocytes had significant positive correlations with the absolute counts of CD8^+^ T lymphocytes, NK cells, and B lymphocytes, while the absolute counts of CD8^+^ T lymphocytes had significant positive correlations with NKT cells. Furthermore, the absolute counts of NK cells were significantly correlated with B lymphocytes (**Figure 4B**).

## 4 Discussion

**Figure 5:**
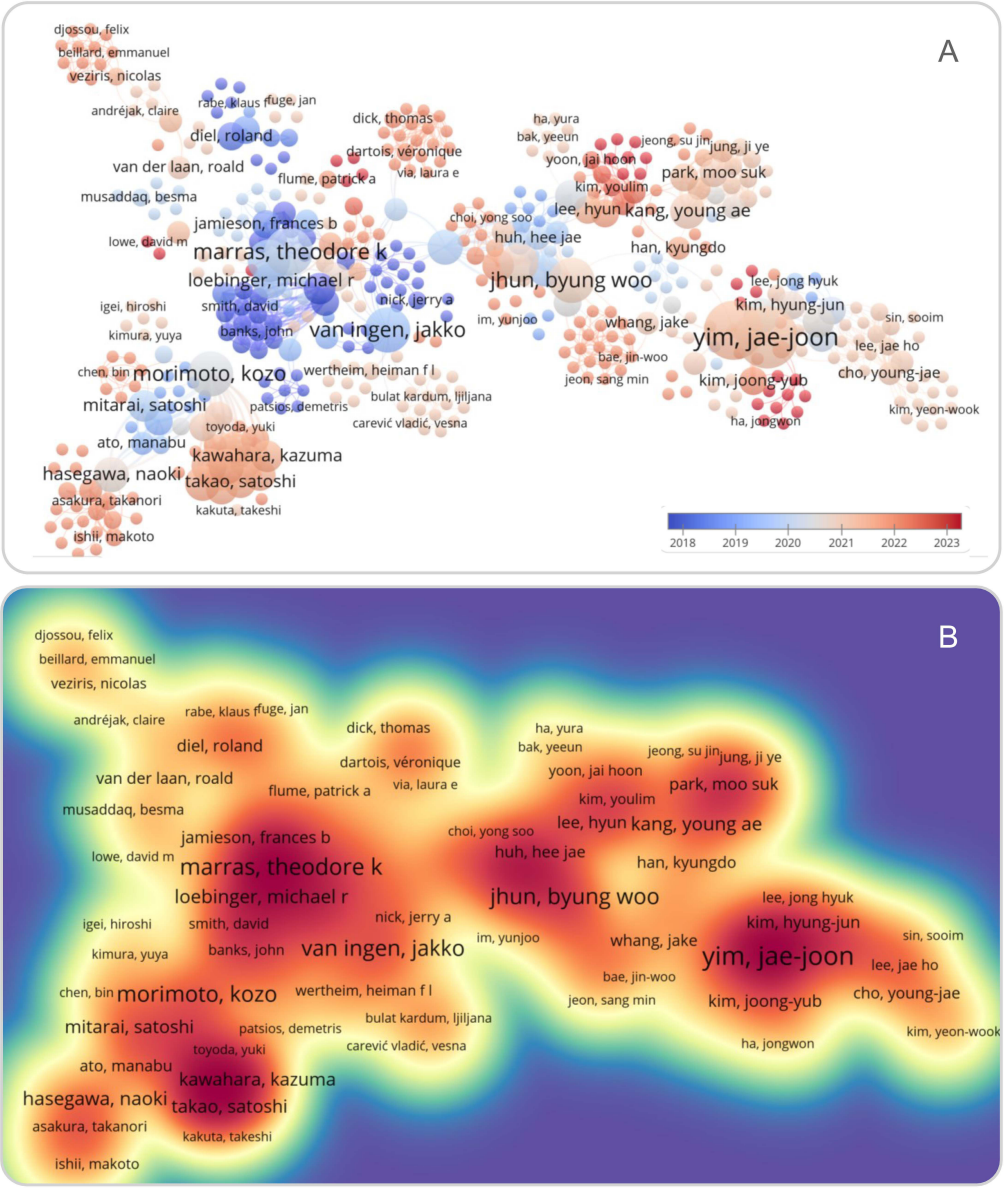
Overlay **(A)** and density **(B)** visualization of the literature related to NTM-PD in PubMed database using VOSviewer Software version 1.6.19 (Leiden University, the Netherlands). The term “((Non-tuberculous Mycobacteria pulmonary disease [title/abstract]) OR (NTM-PD [title/abstract]))” was used to search the literature associated with NTM-PD in the PubMed database. In the overlay visualization, literature published between 2009 and 2023 was shown in blue to red. The density visualization showed citation strength in light yellow to red.

With the improvement of people’s understanding of NTM-PD and the progress of diagnosis and treatment technologies, the detection rate of NTM is increasing. However, the increasing number of drug-resistant strains of NTM has gradually become a new challenge for diagnosing and treating NTM-PD. NTM species also exhibit geographical variation. For example, it has been reported that *M. avium* complex is the most common cause of NTM-PD in Europe and the USA (Hoefsloot et al., 2013). Simultaneously, *M. abscessus* is the second most common cause of NTM-PD in the USA. Additionally, *M. kansasii*, *M. malmoense*, and *M. xenopi* have become the second most common cause of NTM-PD in some European countries and Canada (Griffith et al., 1993; Wassilew et al., 2016). In recent years, more and more attention has been paid to the clinical characteristics and drug resistance data of patients with NTM-PD (**Figure 5**). However, the incidence of NTM-PD in Beijing, the clinical and immunological features of patients with NTM-PD, and the rate of drug resistance are still poorly understood. Therefore, this study was conducted to analyze the incidence, clinical and immunological characteristics of patients with NTM-PD and the drug resistance rate of NTM strains in a tertiary hospital in Beijing from 2015 to 2021.

This study found an increase in the number of diagnosed patients with NTM-PD in Beijing in recent years. There was no correlation between the NTM strains and age, but the incidence of NTM-PD was higher among male patients (58.52%) than female patients (41.48%), which is consistent with a previous study conducted in South Korea (Kim et al., 2023b). Our investigation also revealed that the occurrence of NTM-PD varied among distinct age groups. Strikingly, middle-aged and older adults had a notably higher incidence of NTM-PD compared to their younger counterparts, with a greater prevalence observed in elderly males. These results provide evidence suggesting that aging males are more vulnerable to NTM infection, consistent with previous findings (Jhun et al., 2020; Wang et al., 2022; Alkarni et al., 2023).

In this study, the number of patients with NTM-PD combined with bronchiectasis was relatively high (24.1%). It has been reported that emphysema or bronchiectasis may be associated with alpha-1 antitrypsin (AAT) deficiency in NTM-PD patients (Bai et al., 2019). Interestingly, AAT has been demonstrated to facilitate macrophage-mediated control of intracellular NTM infection by promoting both phagolysosomal fusion and autophagic responses (Bai et al., 2019). Moreover, AAT deficiency is the primary genetic susceptibility factor for the development of COPD (Strange, 2020). Our results indicated that patients with COPD were susceptible to NTM-PD (17.2%), which was consistent with a previous study (Provoost et al., 2018). It might be associated with local airway inflammation, tissue destruction, airway remodeling, small airway stenosis, obstruction, and mucus blockage in COPD patients. In addition, because mycobacteria readily attack the compromised immune system, patients with a history of cancer, diabetes, and immune disorders were likely to be at high risk for NTM-PD. On the other hand, using immunosuppressants and high blood glucose can lead to cell-mediated immune destruction, making these patients more susceptible to NTM-PD (Henkle and Winthrop, 2015).

Cough and sputum (89.70%) were the main clinical manifestations of NTM-PD in this study, followed by hemoptysis (29.9%). Patients with predominant lesion types of bronchiectasis and nodules were found to be more susceptible to NTM-PD, followed by the presence of predominant cavitary lesions, with thin-walled cavities accounting for 90.91% of cases. Symptoms and imaging features of NTM-PD were similar to those of PTB, as previous studies have shown (Hu et al., 2019; Garcia et al., 2022). However, clinical differentiation between PTB and NTM-PD depends on bacteriology and molecular biology. In contrast, clinical symptoms and imaging manifestations of NTM-PD are atypical and difficult to distinguish from PTB. Cavity formation is a sign of disease progression. In this study, we identified cavitary lesions in 33 patients with NTM-PD through CT findings. Among these cases, only 3 (9.1%) showed thick-walled cavitary lesions, which were caused by lung disease resulting from *M. intracellulare*, while the remaining 30 cases showed thin-walled cavitary lesions (90.9%). Patients with NTM-PD with cavitary lesions require prolonged and intensive therapy. Analyzing the location, size, number, and luminal wall thickness of cavitary lesions is critical for determining the prognosis of the disease and the need for additional surgical intervention (Jhun et al., 2020; Kim et al., 2023a). As a result, extensively studying the imaging features of bronchiectasis, thin-walled cavities, and solid lesions is crucial for early detection, diagnosis, treatment, and prognosis of NTM-PD.

NTM is known for its universal drug resistance, and the therapy of NTM-PD is a great challenge. This study identified 23 isolates from 87 NTM-PD patients with NTM strain records. The DST results indicated that *M. abscessus* and *M. avium* were resistant to almost all the anti-TB drugs used in the DST, and over half of the *M. intracellulare* and *M. avium*-intracellulare complex isolates were resistant to anti-TB drugs according to DST results. These findings suggest that there is a high prevalence of drug resistance within NTM strains, particularly to KM, CPM, AMK, and PAS (Gopalaswamy et al., 2020; Park et al., 2022). Resistance to other commonly used antibiotics like INH and macrolides such as AZM also appears to be relatively high. These results emphasize the importance of monitoring antibiotic susceptibility closely in NTM-PD patients and customizing treatment based on the individual patient’s specific strain and antibiotic resistance profile. The drug resistance of the above NTM can be attributed to the natural protective function of the mycobacterial cell wall, which makes it difficult for antibiotics to penetrate mycobacteria, resulting in mycobacteria resistant to many antibiotics (Oh et al., 2017). Furthermore, the efflux pump, biofilm formation, and resistance mutations of NTM are mechanisms essential for protecting the bacteria from elimination by host immune cells and make NTM resistant to anti-TB drugs (Su et al., 2017; Corona et al., 2022; Mudde et al., 2022; Ratna and Daniel, 2023). Additionally, the drug resistance spectra of different NTM species were different. In our study, *M. abscessus*, *M. avium*, and *M. xenopi* showed relatively high drug resistance rates, while *M. kansasii* had relatively low rates of resistance. In addition, *M. avium* and *M. intracellulare* showed different drug resistance. *M. avium* had higher resistance rates to INH, SM, RFP, EMB, KM, CPM, AMK, LVFX, and PAS compared to *M. intracellulare*, indicating a higher drug resistance rate of *M. avium* than that of *M. intracellulare.* In contrast, Wang et al. found that *M. intracellulare* had a higher resistance rate to most of the antimicrobials tested than *M. avium* (Wang et al., 2021). This variability may stem from differences in sample size, detection methods, and regional differences as noted by Wang et al.

Studies have shown that the innate and adaptive immune cells play critical roles in fighting against NTM infections (Behar, 2013). Innate immune cells such as NK cells, macrophages, and dendritic cells (DCs) can eliminate and kill NTM in the early stages of infection by phagocytosis and granzyme (Cruz-Aguilar et al., 2021). Additionally, cellular immunity, mediated by CD4^+^ and CD8^+^ T lymphocytes, promotes the production of various cytokines and chemokines that significantly prevent and control NTM (Abebe, 2012). In this study, we found that the absolute counts of total T lymphocytes, CD4^+^ T lymphocytes, CD8^+^ T lymphocytes, NK cells, and total B lymphocytes in NTM-PD patients were significantly lower than those in the healthy controls, which is consistent with previous studies (Fleshner et al., 2016; Chai et al., 2022). These findings indicate that the cellular and humoral immunity of patients with NTM-PD is significantly suppressed. This leads to an inability to produce cytokines and antibodies against NTM invasion, ultimately resulting in the occurrence of NTM-PD. In addition, NK and NKT cells are vital to the host’s innate immune defense against NTM infection. They produce IFN-γ and IL-22 to inhibit mycobacterial growth, as evidenced in previous studies (Rocco and Irani, 2011; Lai et al., 2018; Gong et al., 2022; Cheng et al., 2023; Peng et al., 2023). It is worth noting that a decrease in lymphocyte count can be influenced by various factors, such as age. Therefore, it may be valuable to investigate whether there were any differences in age range between NTM-PD patients and control group in future studies.

Moreover, we explored the relationship between the absolute number of innate and adaptive immune cells in the patients with NTM-PD and the healthy controls using PCA and correlation analysis. It was found that total T lymphocytes, CD4^+^ T lymphocytes and CD8^+^ T lymphocytes played a more critical role in the immunity of patients with NTM-PD. Furthermore, there was a robust positive correlation between them. Taken together, these findings suggest that augmenting the counts of total T lymphocytes, CD4^+^ T lymphocytes, and CD8^+^ T lymphocytes in patients with NTM-PD may represent a viable strategy to prevent disease deterioration and hinder disease progression.

NTM-PD is becoming increasingly prevalent annually, especially in the immunocompromised population. To address this global issue, further epidemiological clinical and laboratory research is required to provide a robust theoretical and practical framework for early detection, rapid diagnosis, and effective treatment. Fortunately, with the rapid advancements in molecular biology techniques, an expanding number of NTM strains, including their phenotypic and genotypic drug susceptibility, have been identified and analyzed, paving the way for appropriate and clinically effective treatment. However, zoonotic tuberculosis (TB) testing data from 194 countries worldwide revealed that surveillance data on zoonotic TB was deficient in up to 89.9% of the 119 WHO signatory countries (de Macedo Couto et al., 2022). To combat this issue globally, we recommend that all countries incorporate the “One Health” strategy into their TB prevention and treatment agendas, which focuses on TB prevention and control from human, animal, and environmental perspectives for a truly comprehensive approach to combatting TB (Bikom et al., 2022; Zhang et al., 2022).

The One Health approach, which recognizes the interconnectedness of human, animal, and environmental health, is relevant in understanding and addressing NTM-PD prevention and treatment strategies (Zinsstag et al., 2018; Banerjee and van der Heijden, 2023). Firstly, NTM-PD is caused by environmental NTMs that have been found in soil, dust, water, and even in animals, which highlights the importance of environmental and animal health in NTM-PD prevention (Lopeman et al., 2019; To et al., 2020). Measures such as better sanitation, proper disposal of animal waste, and the reduction of environmental pollution can potentially reduce the risk of NTM-PD transmission (Pavlik et al., 2022). Secondly, the study found that impaired immune function is a high-risk factor for NTM-PD. This highlights the importance of considering human health factors in NTM-PD prevention and treatment. Improving the overall health of individuals, including addressing underlying medical conditions and promoting healthy lifestyles, can potentially improve immune function and reduce the risk of NTM-PD (Gompo et al., 2020). Thirdly, the study also identified high levels of drug resistance in NTM-PD patients, which highlights the need for collaboration between human and animal health experts in developing antimicrobial stewardship programs (Rice, 2018; Twabi et al., 2021). Such programs can promote responsible use of antibiotics to minimize the emergence and spread of drug resistant NTM strains.

Based on the findings of this study, utilizing the One Health approach, the prevention and control strategies for NTM-PD should focus on improving environmental hygiene, reducing exposure to NTM in communities, increasing awareness and monitoring of high-risk factors, improving diagnosis and treatment of NTM-PD, and enhancing the immune function of NTM-PD patients through immunomodulatory therapy. Additionally, there should be more collaboration between the human health, animal health, and environmental sectors to prevent and control the spread of NTM-PD.

The present study has several limitations: (1) The phenotype DST did not account for the newly developed drugs; (2) The study only detected lymphocyte subsets without conducting further analysis of their functional cytokines; (3) The NTM-PD patients and healthy individuals were recruited from a single center, rather than multiple centers. To better comprehend the epidemiological characteristics of NTM-PD from cellular and genetic perspectives and formulate effective prevention and control policies in Beijing, it is imperative to conduct a larger, multi-center study in the future.

## 5 Conclusions

In this study, we investigated the clinical and immunological features of patients with NTM-PD in a tertiary hospital in Beijing between 2015 and 2021. The results of this study showed a gradual rise in the incidence of NTM-PD in Beijing over the past seven years, with the primary causative agents being *M. intracellulare* and *M. abscessus*. Additionally, individuals with bronchitis and COPD were more prone to developing NTM-PD. Furthermore, the study revealed that NTM-PD patients exhibit non-specific clinical symptoms, high rates of drug resistance, thin-walled cavity damage on imaging, concomitant bronchiectasis, and significantly lower absolute numbers of innate and adaptive immune cells. These findings provide novel insights into incidence, clinical and immunological features of patients with NTM-PD in Beijing, which support the development of new strategies for early diagnosis and treatment based on drug resistance and absolute counts of immune cells, as well as the implementation of the “One Health” approach to NTM-PD prevention and treatment.

## Data availability statement

The original contributions presented in the study are included in the article/supplementary material. Further inquiries can be directed to the corresponding authors.

## Ethics statement

The studies involving human participants were reviewed and approved by Medical Ethics Committee of the Eighth Medical Center of the PLA General Hospital; Approval Code: 202205311006; Approval Date: 2022-05-25. The patients/participants provided their written informed consent to participate in this study. Written informed consent was obtained from the individual(s) for the publication of any potentially identifiable images or data included in this article.

## Author contributions

Conceptualization: JQL and WG; Data curation: QL, JD, HA, XL, DG, and JBL; Formal analysis: QL, JD, HA, WG, and JQL; Funding acquisition: JQL; Methodology: QL, JD, HA, XL, and DG; Software: QL and WG; Writing - original draft: QL and JD; Writing - review & editing: WG and JQL. All authors contributed to the article and approved the submitted version.

## References

[B1] Abebe F. (2012). Is interferon-gamma the right marker for bacille calmette-guerin-induced immune protection? the missing link in our understanding of tuberculosis immunology. Clin. Exp. Immunol. 169, 213–219. doi: 10.1111/j.1365-2249.2012.04614.x 22861360 PMC3444997

[B2] Alkarni M. Lipman M. Lowe D. M. (2023). The roles of neutrophils in non-tuberculous mycobacterial pulmonary disease. Ann. Clin. Microbiol. Antimicrob. 22, 14. doi: 10.1186/s12941-023-00562-6 36800956 PMC9938600

[B3] Association., T.B.o.C.M (2020). Guidelines for the diagnosis and treatment of nontuberculous mycobacterial disease (2020 edition). Chin. J. Tuberculosis. Respir. 43, 918–946. doi: 10.3760/cma.j.cn112147-20200508-00570

[B4] Bai X. Bai A. Honda J. R. Eichstaedt C. Musheyev A. Feng Z.. (2019). Alpha-1-Antitrypsin enhances primary human macrophage immunity against non-tuberculous mycobacteria. Front. Immunol. 10. doi: 10.3389/fimmu.2019.01417 PMC6606736 31293581

[B5] Banerjee S. van der Heijden M. G. A. (2023). Soil microbiomes and one health. Nat. Rev. Microbiol. 21, 6–20. doi: 10.1038/s41579-022-00779-w 35999468

[B6] Behar S. M. (2013). Antigen-specific CD8(+) T cells and protective immunity to tuberculosis. Adv. Exp. Med. Biol. 783, 141–163. doi: 10.1007/978-1-4614-6111-1_8 23468108 PMC5784412

[B7] Bikom P. M. Nwankwo I. O. Nwanta J. A. (2022). Prevalence and retrospective insight on tuberculosis in human patients in cross river state, nigeria; one health approach to its control. Curr. Microbiol. 79, 345. doi: 10.1007/s00284-022-03046-6 36209340

[B8] Budong C. Hongjun L. Hui C. (2021). Imaging diagnostic criteria of non-tuberculous mycobacterial lung disease. Chin. Res. Hospitals. 8, 6.

[B9] Chai J. Han X. Mei Q. Liu T. Walline J. H. Xu J.. (2022). Clinical characteristics and mortality of non-tuberculous mycobacterial infection in immunocompromised vs. immunocompetent hosts. Front. Med. (Lausanne). 9. doi: 10.3389/fmed.2022.884446 PMC9159854 35665363

[B10] Cheng L. P. Chen S. H. Lou H. Gui X. W. Shen X. N. Cao J.. (2022). Factors associated with treatment outcome in patients with nontuberculous mycobacterial pulmonary disease: a Large population-based retrospective cohort study in shanghai. Trop. Med. Infect. Dis. 7(2):27. doi: 10.3390/tropicalmed7020027 35202222 PMC8876156

[B11] Cheng P. Jiang F. Wang G. Wang J. Xue Y. Wang L.. (2023). Bioinformatics analysis and consistency verification of a novel tuberculosis vaccine candidate HP13138PB. Front. Immunol. 14. doi: 10.3389/fimmu.2023.1102578 PMC9942524 36825009

[B12] Clarke C. Kerr T. J. Warren R. M. Kleynhans L. Miller M. A. Goosen W. J. (2022). Identification and characterisation of nontuberculous mycobacteria in African buffaloes (Syncerus caffer), south Africa. Microorganisms 10(9):1861. doi: 10.3390/microorganisms10091861 36144463 PMC9503067

[B13] CLSI (2011). Susceptibility testing of mycobacteria, nocardia spp., and other aerobic actinomycetes Vol. 2 (Wayne: Clinical and Laboratory Standards Institute). 31339680

[B14] Corona P. Ibba R. Piras S. Molicotti P. Bua A. Carta A. (2022). Quinoxaline-based efflux pump inhibitors restore drug susceptibility in drug-resistant nontuberculous mycobacteria. Arch. Pharm. (Weinheim). 355, e2100492. doi: 10.1002/ardp.202100492 35532283

[B15] Cruz-Aguilar M. Castillo-Rodal A. I. Arredondo-Hernández R. López-Vidal Y. (2021). Non-tuberculous mycobacteria immunopathogenesis: closer than they appear. a prime of innate immunity trade-off and NTM ways into virulence. Scand. J. Immunol. 94, e13035. doi: 10.1111/sji.13035 33655533 PMC9285547

[B16] Daley C. L. Iaccarino J. M. Lange C. Cambau E. Wallace R. J. Jr. Andrejak C.. (2020). Treatment of nontuberculous mycobacterial pulmonary disease: an official ATS/ERS/ESCMID/IDSA clinical practice guideline. Clin. Infect. Dis. 71, e1–e36. doi: 10.1093/cid/ciaa241 32628747 PMC7768748

[B17] de Macedo Couto R. Santana G. O. Ranzani O. T. Waldman E. A. (2022). One health and surveillance of zoonotic tuberculosis in selected low-income, middle-income and high-income countries: a systematic review. PloS Negl. Trop. Dis. 16, e0010428. doi: 10.1371/journal.pntd.0010428 35666731 PMC9203019

[B18] Diel R. Jacob J. Lampenius N. Loebinger M. Nienhaus A. Rabe K. F.. (2017). Burden of non-tuberculous mycobacterial pulmonary disease in Germany. Eur. Respir. J. 49, 1602109. doi: 10.1183/13993003.02109-2016 28446559

[B19] Erkyihun G. A. Alemayehu M. B. (2022). One health approach for the control of zoonotic diseases. J. Zoonoses. 2(1):37. doi: 10.15212/zoonoses-2022-0037

[B20] Falkinham J. O. 3rd (2002). Nontuberculous mycobacteria in the environment. Clin. Chest. Med. 23, 529–551. doi: 10.1016/s0272-5231(02)00014-x 12370991

[B21] Fleshner M. Olivier K. N. Shaw P. A. Adjemian J. Strollo S. Claypool R. J.. (2016). Mortality among patients with pulmonary non-tuberculous mycobacteria disease. Int. J. Tuberc. Lung Dis. 20, 582–587. doi: 10.5588/ijtld.15.0807 27084809 PMC6660916

[B22] Garcia B. Wilmskoetter J. Grady A. Mingora C. Dorman S. Flume P. (2022). Chest computed tomography features of nontuberculous mycobacterial pulmonary disease versus asymptomatic colonization: a cross-sectional cohort study. J. Thorac. Imaging 37, 140–145. doi: 10.1097/RTI.0000000000000610 34292274

[B23] Gompo T. R. Shrestha A. Ranjit E. Gautam B. Ale K. Shrestha S.. (2020). Risk factors of tuberculosis in human and its association with cattle TB in Nepal: a one health approach. One Health 10, 100156. doi: 10.1016/j.onehlt.2020.100156 33117873 PMC7582213

[B24] Gong W. Pan C. Cheng P. Wang J. Zhao G. Wu X. (2022). Peptide-based vaccines for tuberculosis. Front. Immunol. 13. doi: 10.3389/fimmu.2022.830497 PMC8841753 35173740

[B25] Gopalaswamy R. Shanmugam S. Mondal R. Subbian S. (2020). Of tuberculosis and non-tuberculous mycobacterial infections - a comparative analysis of epidemiology, diagnosis and treatment. J. BioMed. Sci. 27, 74. doi: 10.1186/s12929-020-00667-6 32552732 PMC7297667

[B26] Griffith D. E. Girard W. M. Wallace R. J. Jr. (1993). Clinical features of pulmonary disease caused by rapidly growing mycobacteria. an analysis of 154 patients. Am. Rev. Respir. Dis. 147, 1271–1278. doi: 10.1164/ajrccm/147.5.1271 8484642

[B27] Henkle E. Winthrop K. L. (2015). Nontuberculous mycobacteria infections in immunosuppressed hosts. Clin. Chest. Med. 36, 91–99. doi: 10.1016/j.ccm.2014.11.002 25676522 PMC4710582

[B28] Hoefsloot W. van Ingen J. Andrejak C. Angeby K. Bauriaud R. Bemer P.. (2013). The geographic diversity of nontuberculous mycobacteria isolated from pulmonary samples: an NTM-NET collaborative study. Eur. Respir. J. 42, 1604–1613. doi: 10.1183/09031936.00149212 23598956

[B29] Hu C. Huang L. Cai M. Wang W. Shi X. Chen W. (2019). Characterization of non-tuberculous mycobacterial pulmonary disease in nanjing district of China. BMC Infect. Dis. 19, 764. doi: 10.1186/s12879-019-4412-6 31477038 PMC6719376

[B30] Jeon D. (2019). Infection source and epidemiology of nontuberculous mycobacterial lung disease. Tuberc. Respir. Dis. 82, 94–101. doi: 10.4046/trd.2018.0026 PMC6435933 30302953

[B31] Jhun B. W. Moon S. M. Jeon K. Kwon O. J. Yoo H. Carriere K. C.. (2020). Prognostic factors associated with long-term mortality in 1445 patients with nontuberculous mycobacterial pulmonary disease: a 15-year follow-up study. Eur. Respir. J. 55(1):1900798. doi: 10.1183/13993003.00798-2019 31619468

[B32] Katale B. Z. Mbugi E. V. Botha L. Keyyu J. D. Kendall S. Dockrell H. M.. (2014). Species diversity of non-tuberculous mycobacteria isolated from humans, livestock and wildlife in the Serengeti ecosystem, Tanzania. BMC Infect. Dis. 14, 616. doi: 10.1186/s12879-014-0616-y 25403612 PMC4239340

[B33] Kim J. Y. Choi Y. Park J. Goo J. M. Kim T. S. Seong M. W.. (2023a). Impact of treatment on long-term survival of patients with mycobacterium avium complex pulmonary disease. Clin. Infect. Dis. doi: 10.1093/cid/ciad108 36861203

[B34] Kim J. Y. Han A. Lee H. Ha J. Lee K. W. Suh K. S.. (2023b). The clinical course and prognosis of patients with nontuberculous mycobacterial pulmonary disease after solid organ transplantation. J. Korean. Med. Sci. 38, e46. doi: 10.3346/jkms.2023.38.e46 36786088 PMC9925332

[B35] Lai H. C. Chang C. J. Lin C. S. Wu T. R. Hsu Y. J. Wu T. S.. (2018). NK cell-derived IFN-gamma protects against nontuberculous mycobacterial lung infection. J. Immunol. 201, 1478–1490. doi: 10.4049/jimmunol.1800123 30061197

[B36] Liang J. An H. Zhou J. Liu Y. Xiang G. Liu Y.. (2021). Exploratory development of PCR-fluorescent probes in rapid detection of mutations associated with extensively drug-resistant tuberculosis. Eur. J. Clin. Microbiol. Infect. Dis. 40, 1851–1861. doi: 10.1007/s10096-021-04236-z 33792806

[B37] Lopeman R. C. Harrison J. Desai M. Cox J. A. G. (2019). Mycobacterium abscessus: environmental bacterium turned clinical nightmare. Microorganisms 7, 90. doi: 10.3390/microorganisms7030090 30909391 PMC6463083

[B38] Lou H. Zou A. Shen X. Fang Y. Sun Q. Zhang F.. (2023). Clinical features of nontuberculous mycobacterial pulmonary disease in the Yangtze river delta of China: a single-center, retrospective, observational study. Trop. Med. Infect. Dis. 8(1):50. doi: 10.3390/tropicalmed8010050 36668957 PMC9861733

[B39] Morimoto K. Iwai K. Uchimura K. Okumura M. Yoshiyama T. Yoshimori K.. (2014). A steady increase in nontuberculous mycobacteriosis mortality and estimated prevalence in Japan. Ann. Am. Thorac. Soc. 11, 1–8. doi: 10.1513/AnnalsATS.201303-067OC 24102151

[B40] Mortaz E. Adcock I. M. Barnes P. J. (2014). Sarcoidosis: role of non-tuberculosis mycobacteria and mycobacterium tuberculosis. Int. J. Mycobacteriol. 3, 225–229. doi: 10.1016/j.ijmyco.2014.10.008 26786620

[B41] Mudde S. E. Schildkraut J. A. Ammerman N. C. de Vogel C. P. de Steenwinkel J. E. M. van Ingen J.. (2022). Unraveling antibiotic resistance mechanisms in mycobacterium abscessus: the potential role of efflux pumps. J. Glob. Antimicrob. Resist. 31, 345–352. doi: 10.1016/j.jgar.2022.10.015 36347496

[B42] Oh T. S. Kim Y. J. Kang H. Y. Kim C. K. Cho S. Y. Lee H. J. (2017). RNA Expression analysis of efflux pump genes in clinical isolates of multidrug-resistant and extensively drug-resistant mycobacterium tuberculosis in south Korea. Infect. Genet. Evol. 49, 111–115. doi: 10.1016/j.meegid.2017.01.002 28062386

[B43] Park Y. Kim C. Y. Park M. S. Kim Y. S. Chang J. Kang Y. A. (2020). Age- and sex-related characteristics of the increasing trend of nontuberculous mycobacteria pulmonary disease in a tertiary hospital in south Korea from 2006 to 2016. Korean. J. Intern. Med. 35, 1424–1431. doi: 10.3904/kjim.2019.395 32550717 PMC7652645

[B44] Park H. E. Lee W. Choi S. Jung M. Shin M. K. Shin S. J. (2022). Modulating macrophage function to reinforce host innate resistance against mycobacterium avium complex infection. Front. Immunol. 13. doi: 10.3389/fimmu.2022.931876 PMC9730288 36505429

[B45] Pavlik I. Ulmann V. Falkinham J. O. 3rd. (2022). Nontuberculous mycobacteria: ecology and impact on animal and human health. Microorganisms 10(8):1516. doi: 10.3390/microorganisms10081516 35893574 PMC9332762

[B46] Peng C. Tang F. Wang J. Cheng P. Wang L. Gong W. (2023). Immunoinformatic-based multi-epitope vaccine design for co-infection of *Mycobacterium tuberculosis* and SARS-CoV-2. J. Pers. Med. 13, 116. doi: 10.3390/jpm13010116 36675777 PMC9863242

[B47] Prevots D. R. Marras T. K. (2015). Epidemiology of human pulmonary infection with nontuberculous mycobacteria: a review. Clin. Chest. Med. 36, 13–34. doi: 10.1016/j.ccm.2014.10.002 25676516 PMC4332564

[B48] Provoost J. Valour F. Gamondes D. Roux S. Freymond N. Perrot E.. (2018). A retrospective study of factors associated with treatment decision for nontuberculous mycobacterial lung disease in adults without altered systemic immunity. BMC Infect. Dis. 18, 659. doi: 10.1186/s12879-018-3559-x 30547753 PMC6295085

[B49] Ratna S. Daniel J. (2023). Stress-induced non-replicating mycobacterium smegmatis incorporates exogenous fatty acids into glycopeptidolipids. Microb. Pathog. 174, 105943. doi: 10.1016/j.micpath.2022.105943 36502992

[B50] Rice L. B. (2018). Antimicrobial stewardship and antimicrobial resistance. Med. Clin. North Am. 102, 805–818. doi: 10.1016/j.mcna.2018.04.004 30126572

[B51] Ringshausen F. C. Wagner D. de Roux A. Diel R. Hohmann D. Hickstein L.. (2016). Prevalence of nontuberculous mycobacterial pulmonary disease, Germany, 2009-2014. Emerg. Infect. Dis. 22, 1102–1105. doi: 10.3201/eid2206.151642 27191473 PMC4880102

[B52] Rocco J. M. Irani V. R. (2011). Mycobacterium avium and modulation of the host macrophage immune mechanisms. Int. J. Tuberc. Lung Dis. 15, 447–452. doi: 10.5588/ijtld.09.0695 21396201

[B53] Schildkraut J. A. Zweijpfenning S. M. H. Nap M. He K. Dacheva E. Overbeek J.. (2021). The epidemiology of nontuberculous mycobacterial pulmonary disease in the Netherlands. ERJ. Open Res. 7(3):00207-2021. doi: 10.1183/23120541.00207-2021 34262970 PMC8273392

[B54] Sharma S. K. Upadhyay V. (2020). Epidemiology, diagnosis & treatment of non-tuberculous mycobacterial diseases. Indian J. Med. Res. 152, 185–226. doi: 10.4103/ijmr.IJMR_902_20 33107481 PMC7881820

[B55] Strange C. (2020). Alpha-1 antitrypsin deficiency associated COPD. Clin. Chest. Med. 41, 339–345. doi: 10.1016/j.ccm.2020.05.003 32800189

[B56] Su K. Y. Yan B. S. Chiu H. C. Yu C. J. Chang S. Y. Jou R.. (2017). Rapid sputum multiplex detection of the m. tuberculosis complex (MTBC) and resistance mutations for eight antibiotics by nucleotide MALDI-TOF MS. Sci. Rep. 7, 41486. doi: 10.1038/srep41486 28134321 PMC5278408

[B57] Tan Y. Deng Y. Yan X. Liu F. Tan Y. Wang Q.. (2021). Nontuberculous mycobacterial pulmonary disease and associated risk factors in China: a prospective surveillance study. J. Infect. 83, 46–53. doi: 10.1016/j.jinf.2021.05.019 34048821

[B58] Tan Y. Su B. Shu W. Cai X. Kuang S. Kuang H.. (2018). Epidemiology of pulmonary disease due to nontuberculous mycobacteria in southern China, 2013-2016. BMC Pulm. Med. 18, 168. doi: 10.1186/s12890-018-0728-z 30413193 PMC6230232

[B59] Tingan T. K. Mensah G. I. Agyekum E. B. Amanor I. B. Addo S. O. Ayamdoo Y. I.. (2022). Non-tuberculous mycobacteria, not mycobacterium bovis, are a significant cause of TB-like lesions observed in slaughtered cattle in Ghana. IJID. Reg. 3, 8–14. doi: 10.1016/j.ijregi.2022.02.004 35755480 PMC9216640

[B60] To K. Cao R. Yegiazaryan A. Owens J. Venketaraman V. (2020). General overview of nontuberculous mycobacteria opportunistic pathogens: mycobacterium avium and mycobacterium abscessus. J. Clin. Med. 9(8):2541. doi: 10.3390/jcm9082541 32781595 PMC7463534

[B61] Twabi H. H. Mukoka-Thindwa M. Shani D. Nliwasa M. Corbett E. L. (2021). Non-tuberculous mycobacterial pulmonary disease identified during community-based screening for mycobacterium tuberculosis: a case report. Malawi. Med. J. 33, 65–67. doi: 10.4314/mmj.v33i1.10 34422236 PMC8360289

[B62] van der Laan R. Snabilie A. Obradovic M. (2022). Meeting the challenges of NTM-PD from the perspective of the organism and the disease process: innovations in drug development and delivery. Respir. Res. 23, 376. doi: 10.1186/s12931-022-02299-w 36566170 PMC9789522

[B63] Wang J. Xu H. Wang X. Lan J. (2022). Rapid diagnosis of non-tuberculous mycobacterial pulmonary diseases by metagenomic next-generation sequencing in non-referral hospitals. Front. Cell Infect. Microbiol. 12. doi: 10.3389/fcimb.2022.1083497 PMC9902348 36760234

[B64] Wang W. Yang J. Wu X. Wan B. Wang H. Yu F.. (2021). Difference in drug susceptibility distribution and clinical characteristics between mycobacterium avium and *Mycobacterium intracellulare* lung diseases in shanghai, China. J. Med. Microbiol. 70(5). doi: 10.1099/jmm.0.001358 33999797

[B65] Wassilew N. Hoffmann H. Andrejak C. Lange C. (2016). Pulmonary disease caused by non-tuberculous mycobacteria. Respiration 91, 386–402. doi: 10.1159/000445906 27207809

[B66] Winthrop K. L. Marras T. K. Adjemian J. Zhang H. Wang P. Zhang Q. (2020). Incidence and prevalence of nontuberculous mycobacterial lung disease in a Large U.S. managed care health plan, 2008-2015. Ann. Am. Thorac. Soc. 17, 178–185. doi: 10.1513/AnnalsATS.201804-236OC 31830805 PMC6993793

[B67] Xu J. Li P. Zheng S. Shu W. Pang Y. (2019). Prevalence and risk factors of pulmonary nontuberculous mycobacterial infections in the zhejiang province of China. Epidemiol. Infect. 147, e269. doi: 10.1017/S0950268819001626 31506134 PMC6807301

[B68] Zhang H. Liu M. Fan W. Sun S. Fan X. (2022). The impact of mycobacterium tuberculosis complex in the environment on one health approach. Front. Public Health 10. doi: 10.3389/fpubh.2022.994745 PMC9489838 36159313

[B69] Zhao Y. Pang Y. (2015). Testing procedures for tuberculosis laboratory (Beijing, China: People’s Medical Publishing House).

[B70] Zhou L. Ma C. Xiao T. Li M. Liu H. Zhao X.. (2019). New single gene differential biomarker for mycobacterium tuberculosis complex and non-tuberculosis mycobacteria. Front. Microbiol. 10. doi: 10.3389/fmicb.2019.01887 PMC6700215 31456790

[B71] Zinsstag J. Crump L. Schelling E. Hattendorf J. Maidane Y. O. Ali K. O.. (2018). Climate change and one health. FEMS Microbiol. Lett 365(11):fny085. doi: 10.1093/femsle/fny085 29790983 PMC5963300

